# Micromotion analysis of immediately loaded implants with Titanium and Cobalt‐Chrome superstructures. 3D finite element analysis

**DOI:** 10.1002/cre2.365

**Published:** 2021-05-27

**Authors:** Julio Tobar‐Reyes, Luis Andueza‐Castro, Antonio Jiménez‐Silva, Roger Bustamante‐Plaza, Juan Carvajal‐Herrera

**Affiliations:** ^1^ Department of Oral Rehabilitation, Faculty of Dentistry University of Chile Santiago Chile; ^2^ Facultad de Diseño Pontificia Universidad Católica de Chile Santiago Chile; ^3^ Orthodontic and Orthopaedic Department, Faculty of Dentistry Universidad Andrés Bello Santiago Chile; ^4^ Facultad de Ciencias Físicas y Matemáticas Universidad de Chile Santiago Chile

**Keywords:** dental implants, finite element analysis, motion, osseointegration

## Abstract

**Objective:**

The aim of this study was to evaluate the amount of micromotion of dental implants under immediate loading supported by Titanium (Ti) and Cobalt‐Chrome (Co‐Cr) superstructures.

**Material and methods:**

A model of tridimensional half‐edentulous maxilla with three dental implants was made using the Finite Element Analysis (FEA). Two standard and one zygomatic implants were connected to a superstructure with an elliptic section of 6x 3 mm (mm). Two study models were established. Model A: Titanium (Ti) alloy superstructure; Model B: Cobalt‐Chrome (Co‐Cr) alloy superstructure. To simulate an immediate‐loading situation, a friction coefficient of 0.71 was applied between the implant and the bone surface. An axial load of 252.04 [N] was applied on standard and zygomatic implants.

**Results:**

The Micromotion of dental implants was similar in both superstructure situations. The amount of micromotion was slightly higher in B1 and B3 models (Co‐Cr alloy‐superstructure) compared with A1 and A3 models (Titanium alloy superstructure). The micromotion values in two groups were greater than 150 μm in the incisive region (standard implant) and molar region (zygomatic). In general, the micromotion was higher on the implant that received the load with respect to the other implants. The greater difference was observed when the load was applied on the standard implant A1 (Model A1 = 189.12 μm) compared with standard implant B1(Model B1 = 263.25 μm).

**Conclusions:**

Within the limits of present study, all implants on different load application points showed micromotion; in general, the amount of micromotion was slightly higher in the implants connected with Co‐Cr alloy superstructure.

## 1 INTRODUCTION

Dental implants have been used for the last decades for anchoring dental prostheses in the treatment of partial and/or total edentulous patients. In severely atrophic edentulous maxilla, a lower quantity and quality of bone exists and there is not enough bone to allow the support of dental implants (Capelli et al., 2007). A maxillary implant‐supported prosthesis design is an alternative that includes four standard implants in the anterior maxilla and two posterior implants in the zygomatic bone (Balshi et al., 2009; Brånemark et al., 2004; Hu et al., 2007; Kreissl et al., 2007; Malevez et al., 2004; Nkenke et al., 2003; Parel et al., 2001; Uchida et al., 2001; Uckan et al., 2005). Zygomatic implants are an alternative that reduces the need for bone graft procedures (Nkenke et al., 2003), offering support that allows primary stability for immediate loading protocols (Margossian et al., 2012).

In patients with atrophic maxilla, immediate loading implant protocol has been a treatment option for the reduction in the treatment time (Atieh et al., 2010; Chen et al., 2004; Chung et al., 2011; Esposito et al., 2007, 2010; Grütter & Belser, 2009). The traditional protocol suggested a healing period to allow osseointegration between bone and interface implant. During this healing period, forces could not be applied; However, evidence suggests that implants can be immediately loaded under some conditions, without interfering with the osseointegration process (Cehreli et al., 2004; Degidi et al., 2003).

Micromotion has been described as one of the most important variables affecting the success of immediate loading in dental implants. Micromotion has been defined as “The relative movement of the surface of the implant relative to the surrounding bone” (Abdul‐Kadir et al., 2008; Cehreli et al., 2004).The evidence available suggests that there is a critical threshold of micromotion above which fibrous encapsulation prevails over osseointegration, causing a disruption of the bone formation process around the implant resulting in formation of a fibrous tissue layer at the bone–implant interface (Abdul‐Kadir et al., 2008; Cehreli et al., 2004). Cameron et al. (1973) and Szmukler‐Moncler et al. (1998) described a tolerance range of micromotion between 50 and 150 μm.

The designs in complete arch‐prostheses supported by implants, consider rigid splinting by a metallic superstructure, allowing biomechanical support to the occlusal loads, distributing them equally and limiting the amount of implant micromovement during implant healing, providing adequate support to the prosthesis (Behnaz et al., 2015; Cehreli et al., 2005; Guichet et al., 2002; Matsuzaka et al., 2007; T.‐M.Wang et al., 2002). The factors related to the superstructure that would influence implants micromotion could be: cross‐sectional superstructure shape (Korioth & Johann, 1999), low elasticity of the continuum of superstructure and the type of material used in the design (Benzing et al., 1995), which would influence the flexural strength and deformation of the superstructure. Nowadays, two of the most used materials to make these superstructures are Cobalt‐Chrome (Co‐Cr) and Titanium alloy (Drago & Howell, 2012).

The purpose of the study was to determine the influence of the type of alloy of the superstructure supported by two standard and zygomatic implants on the micromotion under immediate loading. The amount of micromotion was analyzed using FEA according to Ti‐alloy and Co‐Cr alloy of the superstructure with section of 6 × 3 mm.

## 2 MATERIAL AND METHODS

A 3‐dimensional finite element model of a half maxilla with two standard dental implants (incisive and canine site) and zygomatic implant (molar site) was performed. Two models were established according to the alloy of superstructure. Model A: Titanium (Ti) alloy superstructure; Model B: Cobalt‐Chrome (CoCr) alloy superstructure. Micromotion was assessed in standard and zygomatic implants in both models when loads were applied.

The study was performed in accordance with the principles of the Declaration of Helsinki. The protocol and the informed consent form were approved by the Ethics Committee of the Faculty of Dentistry at the University of Chile (certificate no. 2016/31).

### 2.1 Construction of 3‐dimensional models, implants and superstructures

**FIGURE 1:**
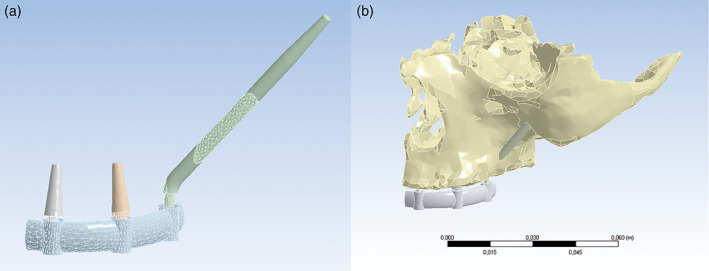
(a) Splinting structure. Standard implants, zygomatic implant and superstructure. (b) Model of a half maxilla with two standard dental implants (incisive and canine site) and zygomatic implant (molar site)

A computed tomography (CT) of an edentulous left maxilla of a 68‐year‐old man was arbitrarily chosen. With this CT a 3‐dimensional FEA human model was constructed with the commercial Mimics® software (Materialize, 1992). Three dental implants were inserted in the site of incisive, canine and molar teeth; two standard implants with a monoblock conical macrodesign, without thread. (4.0 × 10.0 mm) in the incisive site (Implant 1: group A1 and B1) and in the canine site (Implant 2: group A2 and B2) and a zygomatic implant with angled head, monoblock tronco‐conical shape, without thread (3.6 × 33.5 mm) in the molar site (Implant 3: group A3 and B3). A prosthetic superstructure was modeled with s 6 × 3 mm elliptical section; this cross section is used to compensate the interocclusal distance and to perform a rigid splint that prevents flexion of the same (Figure 1a) (Drago & Howell, 2012). Dental implants and supra‐structure were modeled in Autodesk Inventor 2014 software (Autodesk Inc.).

The models were assembled (left maxilla, dental implants and superstructure) (Figure 1b) and the complete geometry was exported in two parts: 1. the modified left maxilla with the holes for dental implants; 2. an assembly with the implants and superstructure. The implants in their coronary portion and prosthetic superstructure were set as a “bond,” referring to a condition in which no relative movement would occur between the components, when loaded with external forces (Freedman et al., 2013, 2015; Ujigawa et al., 2007).

As a model medium is being used, a restriction condition must be applied to the extra movement that simulates the symmetry conditions. For this purpose, a frictionless restriction is assigned to avoid movement in the direction of the X axis. Total restriction of movement is also added to the upper part of the maxilla, ensuring that it is in the furthest parts of the study area (Freedman et al., 2013, 2015; Ujigawa et al., 2007).

The mechanical properties of dental implants and the superstructure were considered as isotropic. The elastic modulus and the Poisson coefficient were obtained from the literature (Table 1) (Geng et al., 2001).

**TABLE 1:** Mechanical properties of the implants used in FEA (30)

Material	Modulus of elasticity [GPa]	Poisson module
Ti_6_Al_4_V	110	0.33
Co‐Cr	218	0.33

Abbreviation: GPa, Gigapascals.

### 2.2 Mesh and assignment of materiality

**FIGURE 2:**
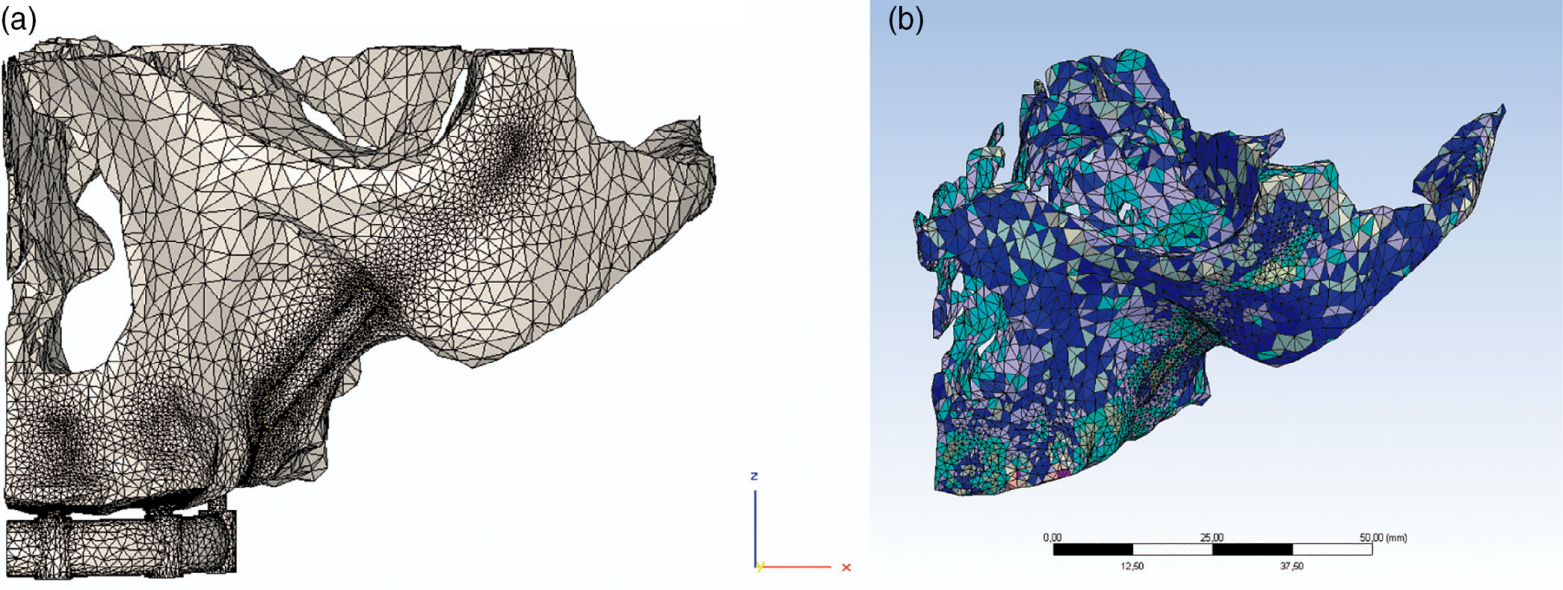
(a) Mesh and assignment of materiality. (b) Mesh with assignment of properties according to bone density of each element. Each color represents a density

The assembled model was transferred to Mimics® software and mesh was performed using second degree tetrahedral elements (Table 2; Figure 2a,b).

**TABLE 2:** Maxillary mesh with elliptical superstructure 6 × 3 mm

Model	Mesh	Maximum size of element	Number of elements
Models A, B	Implants without threads	4 mm in general and 1 mm in the implant face	120.159

*Note*: Maximum sizes and number of elements by mesh.

Abbreviation: mm, Millimeter.

**FIGURE 3:**
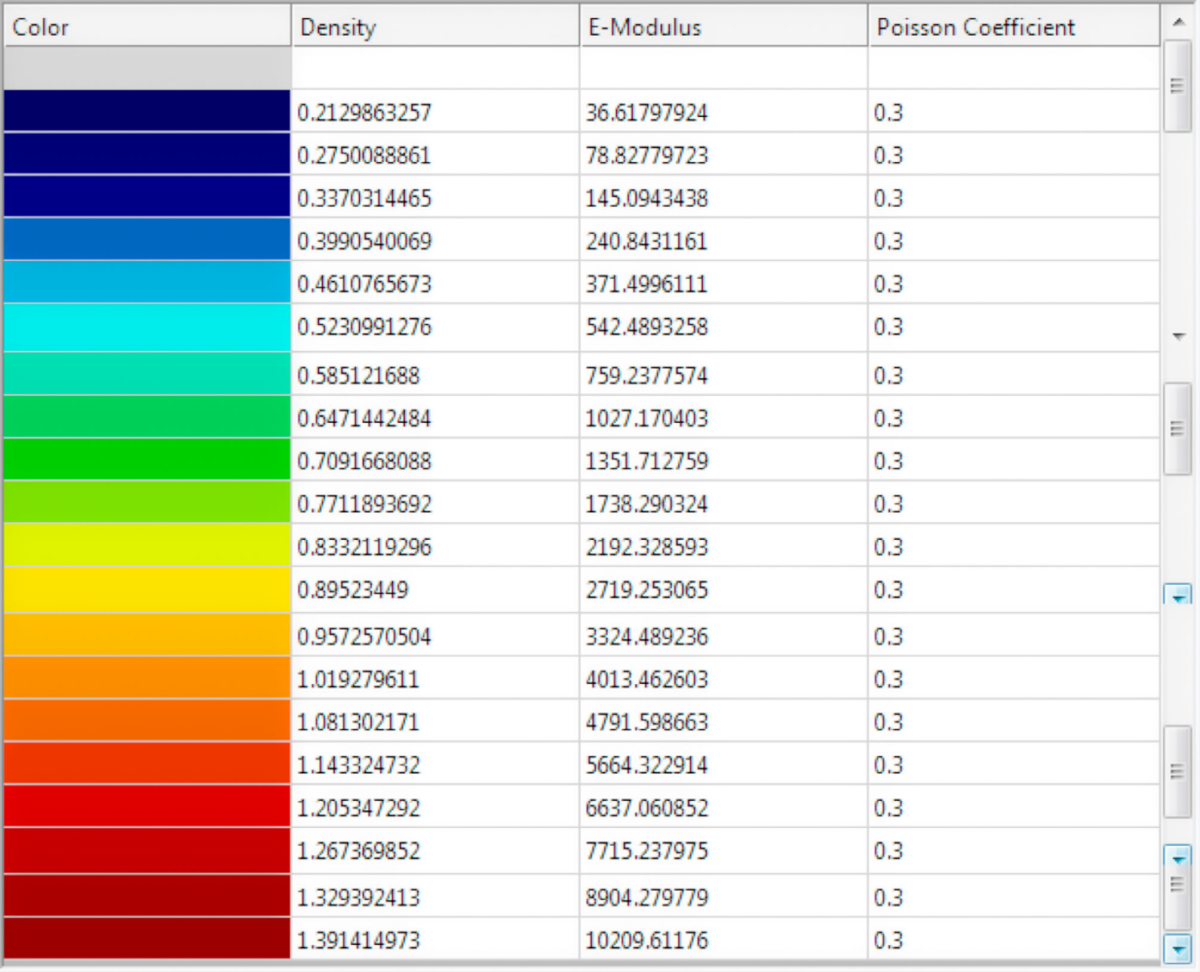
Densities of 20 materials in which the model is divided, along with assigned properties

When the mesh was obtained, the mechanical properties were assigned defining the Young's Modulus and the Poisson's Module, dividing equally the range of Hounsfield Units taken from the CTs into 20 isotropic materials (Figure 3). For the Young's Module, the expression of Carter & Hayes was used (1977), which relates the Modulus of Elasticity to the apparent density from the medical images.

$$E=3790\rho^{3}\;\left(\mathrm{MPa}\right)\;\left(\text{Carter}\&\text{Hayes},1977\right).$$

The apparent density was obtained from the linear approximation of density for the maximum and minimum Hounsfield values from CTs.

$$\rho =0.00004*\mathrm{HU}+0.1185\;\left(\frac{g}{{\mathrm{cm}}^{3}}\right)\;\left(\text{Ishak}\;\mathrm{et}\;\mathrm{al}.,2012\right).$$

The Poisson Module was considered constant for all ranges of Hounsfield Units (Ishak et al., 2012; M. Wang et al., 2013) with a value *υ* = 0.3 (Ishak et al., 2012; M. Wang et al., 2013).

The models obtained from the meshes were transferred to Ansys Workbench software to determine the structural calculation. Once imported, the mesh, its elements and the assignment of materials of the models were verified (Figure 2a,b).

### 2.3 Geometry and contact surfaces

The contact surfaces between the geometries were defined to assign interaction properties to these contacts. Friction contact zones were assigned between dental implant and the maxillary bone to simulate an immediate‐loading situation at the bone‐implant interface. A coefficient of friction of 0.71 was adopted from the average between the friction coefficients of titanium alloy (dental implant) and the cortical bone, equal to 0.65 (Yu et al., 2005) and the coefficient of friction between the titanium implant and trabecular bone, equal to 0.77 (Grant et al., 2007).

### 2.4 Boundary and loading conditions

Micromotion is the relative displacement of the implant in the maxillary bone. It is measured as the maximum node slip between the two surfaces in contact, calculated by subtracting the nodal displacement between the bone and the implant (Berahmani et al., 2017; Winter et al., 2013).

In this study, two models (Model A and Model B) were performed and six situations were established:

**Models A1 and B1:** to simulate occlusal force, a vertical load on dental implant 1 (first standard implant) was applied, located in the incisive site (midline of maxilla).

**Models A2 and B2:** a vertical load was applied on dental implant 2 (second standard implant) located in the canine site.

**FIGURE 4:**
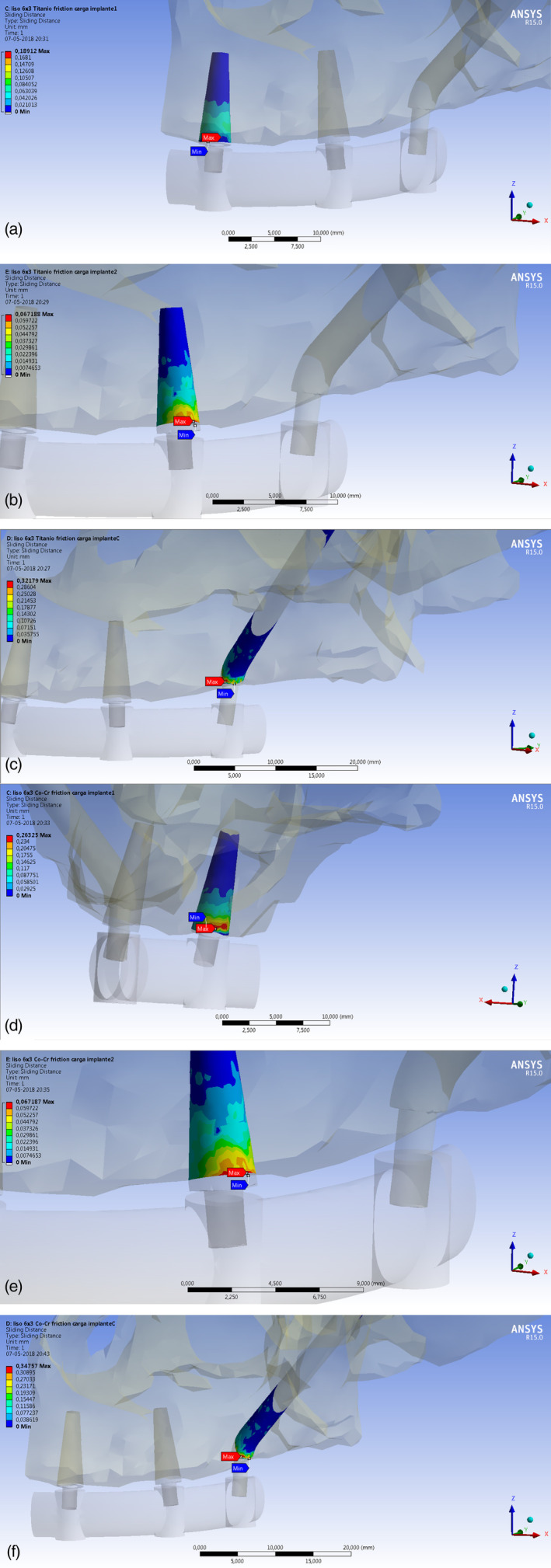
(a)–(c) Micromotion distribution between alveolar bone and implant Models A (Ti) when the occlusal force was applied on dental standard implant 1 (Model A1), standard implant 2 (Model A2), zygomatic implant (Model A3), respectively. (d)–(f) Micromotion distribution between alveolar bone and implant Models B (Co‐Cr) when the occlusal force was applied on dental standard implant 1 (Model B1), standard implant 2 (Model B2), zygomatic implant (Model B3), respectively

**Models A3 and B3:** a vertical load was applied on dental implant 3 or zygomatic implant (Figure 4).

The load applied on the model consisted of a vertical force with a magnitude of 252.04 Newton [N] or 25.71 [Kgf] (global axis was [25, −20, 250] [N]), The simulated occlusal force corresponds to the condition of maximum intercuspation of the teeth, a situation in which the implants receive a vertical compressive load. The magnitude of the applied load was the same for all implants (Geringer et al., 2014).

## 3 RESULTS

In general, when the models were analyzed using FEA, all implants under immediate loading conditions and different load application points showed micromotion, which was similar in dental implants supported by Titanium (Ti) and Cobalt‐Chrome (CoCr) superstructures.

**FIGURE 5:**
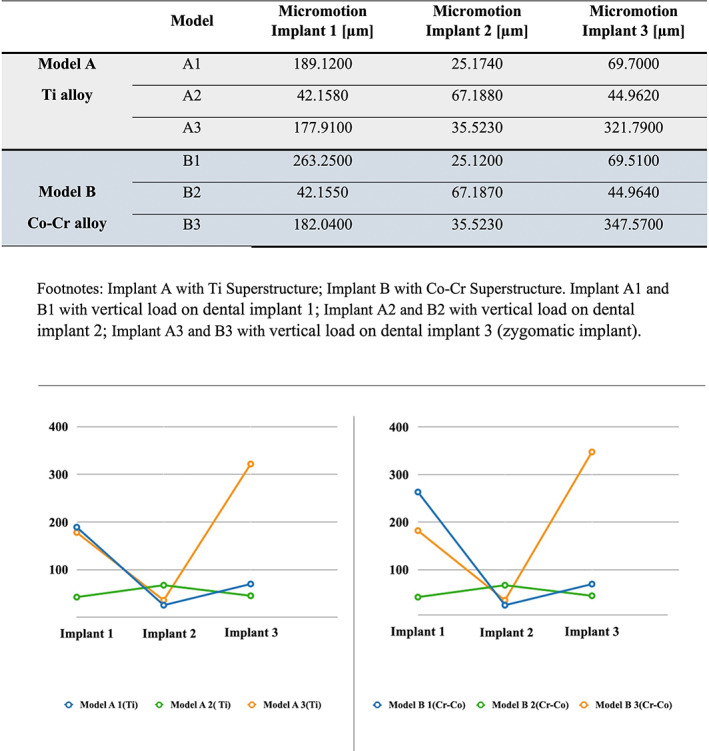
Amount of micromotion observed in the two study groups (Model A and B). The micromotion of each implant is observed

It was observed that implants connected to the CoCr alloy superstructure (Models B1, B2 and B3) obtained slightly higher micromotion values compared to the Titanium alloy (Models A1, A2 and A3). The micromotion was greater in the implant where the load was applied compared to the other implants in all cases studied (Figures 4 and 5). In addition, it was observed that when the load was applied in the molar zone (zygomatic implants or implant 3), there was the greatest amount of micromotion, followed by implant 1 (incisive zone) in both models A3 and B3 (Figure 5).

The amount of micromotion generated in the implant 1 that supported titanium alloy superstructure (model A1) was 189.1200 μm, while its homologous connected with a CoCr alloy superstructure (model B1), was higher by 39.2% (263.2500 μm).

Dental implants located in the canine site (implant 2, models A and B) showed smaller micromotion than other implants, with values ranging from 25 to 67 μm independent of the site of application of the load and the alloy of superstructure (Figures 4 and 5).

When loading was applied in the molar region or implant 3 of the Ti and CoCr alloy (models A3 and B3), an increase in the amount of micromotion was observed in both the implant where load was applied (implant 3) and in implant 1 (Figures 4c,f and 5).

## 4 DISCUSSION

Primary stability is an important variable that could affect the success of immediately loaded implants. The aim of this study was to compare, through FEA, the amount of micromotion in dental implants connected to superstructures of two different alloys (Co‐Cr and Ti) under immediate loading. The results obtained on this study showed that the implant micromotion was similar with both structures, being slightly superior in the Cobalt‐Chromium superstructures alloy (Figure 5).

The result obtained from a 39.2% greater micromotion in the structure of CoCr when applying an occlusal load on implant 1 (incisive zone) and comparing the micromotion generated in the group of Titanium alloy (A1/B1 respectively), would be explained by the greater stiffness of the CoCr alloys with respect to the Titanium, which would generate more Von mises stress in the bone‐implant interface, producing an increase of micromotion. The limited evidence available regarding the distribution of the load in a metallic structure supported by dental implants indicates that the tension of the implant abutment depends significantly on the type of metal alloy (Akça et al., 2002). In addition, the metal structure appeared to be more sensitive to the material stiffness, in agreement with the evidence based on FEA (Spazzin et al., 2011).The FEA analysis performed by Abreu et al., 2010, showed changes in the stress induced by the bar framework, (metal structure) screw neck and implant platform for different bar materials with higher stress levels in the CoCr alloy, suggesting that these components of the system are more sensitive to stiffer materials (Abreu et al., 2010). However, the findings of this study disagree with Benzing et al. (1995) who concluded that the bone‐implant interface would be influenced by the type of alloy metal used in the prosthetic superstructure, showing that the use of alloys with a low module of elasticity would generate more tensions (Benzing et al., 1995).

When the load was applied on the implant 1 (A1 and B1 models), the values of micromotion exceeded 150 μm only on the implants where the load was applied, while the implants located at distance from the load application point showed lower ranges of micromotion. In this regard, the results showed two situations: in the first, the greatest amount of micromotion occurred on the implant where the load was applied. The second situation observed was an increase in micromotion in the implant 1 (incisive area) when the load was applied on the implant 3 (molar area) (Figures 4 and 5). In this study two factors could influence these results; the first, is the absence of threads of the implants that decrease the primary implant stability, according to the published evidence (Chou et al., 2008). As observed by Balshi et al, the use of implants with thread would generate a 30% decrease in displacement between the implant and the bone, compared to implants without threads (Balshi et al., 2009). The second factor is the arbitrary value given in this study to the coefficient of friction between the surface of the implant and the surrounding bone. This coefficient is intended to simulate the insertion torque of an implant, increasing the primary stability of the implants (Oktenoğlu et al., 2001; Sakoh et al., 2006).

The clinical evidence describes a successful osseointegration, with fluctuating implant micromotion range between 50–150 μm (Brunski, 1999; Holmes & Loftus, 1997; Holst et al., 2008; Kawahara et al., 2003; Romanos, 2004; Szmukler‐Moncler et al., 1998; Udofia et al., 2007). The results obtained in this study, showed that in 30% of these results, the micromotion exceeded 150 μm. As for A3 and B3 models, exceeding widely 150 μm when the load was applied in the molar area (zygomatic implants), it is not possible to suggest if this amount of micromotion under immediate loading would imply a potential risk to prevent osseointegration. However, some clinical studies show that the use of zygomatic implants under the immediate loading protocol would have success rates of 98.5% after 5 years of follow‐up in patients with atrophic maxilla (Davó et al., 2013). A tetracortical anchorage and zygomatic bone density would determine adequate primary stability, allowing immediate loading of zygomatic implants (Davó et al., 2007; Nkenke et al., 2003). Kato et al., indicates that the zygomatic bone region does not decrease its density after tooth loss by insertion of the masseter muscle, which subjects this zone to continuous stress, maintaining the osteoblastic activity (Kato et al., 2005). Another explanation for the increased micromotion values, according to studies by Wen et al., showed that zygomatic implants present an increased cantilever, elevating the risk of failure. In addition, they found increased stress generated on these implants over standard implants (Wen et al., 2014).

The limited evidence available in the literature does not permit to draw any definite conclusions. The authors recommend that future research should consider the use of complete maxillary models with the presence of threads in the implants and preload to simulate biomechanical conditions closer to a real clinical situation.

## 5 CONCLUSIONS

In relation to the findings in this study and its limitations we can conclude that:

- All dental implants of this study showed micromotion under the different load conditions, either splinted with Ti or CoCr metal superstructure.
- The micromovement of splinted implants with a CoCr metal alloy was slightly greater than the micromovement in the splinted implants with Ti metal alloy.

1. With respect to the micromotion observed in standard and zygomatic implants:

- The micromotion thresholds established in the literature, as a condition of success of the osseointegration, were exceeded when the load was applied directly on implant 1 either with a Ti or CoCr superstructure alloy.
- Implant 2, independent of the point of application of the load and the superstructure alloy, presented values of micromotion compatible with a success condition of osseointegration established in the literature.
- When the load was applied on implant 3 (zygomatic implant), in the Titanium and Cobalt‐Chrome superstructure, the micromotion thresholds were exceeded according to the literature as a successful condition of osseointegration.

## CONFLICT OF INTEREST

The authors declare no conflict of interest.

## AUTHOR CONTRIBUTIONS

**Tobar‐Reyes Julio:** Supervision and manuscript correction. Execution of three‐dimensional anatomical models. Execution ofmodels of materials in Oral Rehabilitation. **Andueza‐Castro Luis:** Supervision manuscript. Execution of three‐dimensional anatomical models. Execution of models of materials in Oral Rehabilitation. **Jiménez‐Silva Antonio:** Manuscript preparation and translation. Assistance in the execution of three‐dimensional anatomical models and the execution of the material models in Oral Rehabilitation. **Bustamante‐Plaza Roger:** Supervision and manuscript correction. Supervision in the use of specialized softwares in Finite Element Modeling. Review of the progress of the study. **Carvajal‐Herrera Juan:** Supervision and manuscript correction. Review of the progress of the study.

## Data Availability

The data that support the findings of this study are available on request from the corresponding author. The data are not publicly available due to privacy or ethical restrictions.
